# Laparoscopic Bilateral Cervicosacropexy and Vaginosacropexy: New Surgical Treatment Option in Women with Pelvic Organ Prolapse and Urinary Incontinence

**DOI:** 10.1089/end.2018.0474

**Published:** 2018-11-08

**Authors:** Sokol Rexhepi, Entela Rexhepi, Martin Stumm, Peter Mallmann, Sebastian Ludwig

**Affiliations:** ^1^ Department of Obstetrics and Gynecology, Hospital Eichstätt, Eichstätt, Germany.; ^2^ Division of Urogynecology and Pelvic Reconstructive Surgery, Department of Obstetrics and Gynecology, University of Cologne, Köln, Germany.

**Keywords:** uterosacral ligaments, mixed urinary incontinence, urgency urinary incontinence, polyvinylidene fluoride, pelvic organ prolapse, cervicosacropexy, vaginosacropexy

## Abstract

***Objective:*** Sacrocolpopexy (SCP) is the gold standard for apical prolapse treatment. However, the technical performance of each SCP is strongly dependent on the surgeon's own discretion and comparison of clinical outcomes with respect to urinary incontinence (UI) is difficult. We developed a comprehensible laparoscopic surgical technique for the treatment of apical prolapse with UI.

***Methods:*** A total of 120 women with UI underwent laparoscopic bilateral SCP for apical prolapse. Thereby, the uterosacral ligaments (USLs) were bilaterally replaced by polyvinylidene fluoride (PVDF) tapes of identical length and shape, which were fixed at defined anatomical landmarks (cervix/vaginal vault and S1).

***Results:*** The restoration of apical vaginal support was achieved in 116 patients (97%); restoration failed in the first 4 patients owing to the use of fast-absorbable sutures. Seventy-eight patients (65%) with mixed and urgency UI symptoms before surgery achieved continence. The mean hospitalization was 3 days; no major complications were observed intraoperatively.

***Conclusion:*** The advantage of laparoscopic cervicosacropexy (laCESA) and laparoscopic vaginosacropexy (laVASA) lies in the comprehensible surgical technique (clearly defined technique) and the minimal amount of material used (no polypropylenes). The possibility of a short operating time and short hospitalization depicts this laparoscopic bilateral USL replacement as one treatment alternative in patients with apical prolapse suffering from UI.

## Introduction

According to Ulmsten, Petros, and DeLancey, stress and urgency urinary incontinence (SUI and UUI, respectively) result from the laxity of the anterior vaginal wall.^1,2^ Defects of the respective holding apparatus of the uterus and the vagina cause the laxity of the anterior vaginal wall and cause prolapse and urinary incontinence (UI).^1^

To treat these defects of the holding apparatus, Jäger and colleagues initially developed a surgical technique to “standardize” the treatment of apical prolapse.^3,4^ The left and the right uterosacral ligaments (USLs) were replaced. While finding the correct lengths of the USL (i.e., the tension of the apical fixation), Jäger and colleagues arrived at the conclusion that this length is between 8.8 and 9.3 cm. The USLs were replaced by polyvinylidene fluoride (PVDF) structures of these lengths, which were fixed at defined fixation sites (cervix or vaginal vault and the prevertebral fascia in front of S1). The shape and amount of material were reduced to minimum. These surgical techniques (abdominal route) were referred to as cervicosacropexy (CESA) and vaginosacropexy.^5,6^ Notably, in addition to the correction of prolapse, the restoration of urinary continence was observed after these procedures.^3,7,8^

As a certified center for minimally invasive gynecological endoscopy, it was our aim to transform the surgical techniques for bilateral USL replacement into a laparoscopic approach (see Supplementary Video available online at www.liebertpub.com/end). In this study, we present the surgical technique and the clinical outcomes of patients with genital prolapse and UI after laparoscopic cervicosacropexy (laCESA) and laparoscopic vaginosacropexy (laVASA).

## Materials and Methods

This retrospective study included women who underwent laparoscopic bilateral USL replacement at a primary care hospital, Hospital Eichstätt, Department of Obstetrics and Gynecology, between March 2013 and March 2016.

As a certified center of the German Society for Minimally Invasive Gynecological Endoscopy, laparoscopy is performed for many gynecological conditions in our hospital. Approval for the development of the laparoscopic approach of CESA and VASA was obtained from our clinical directors through a conference and the ethics committee (Approval No. 11-016). Written informed consent was obtained from all patients before surgery. Approval for the evaluation of patients after surgery was obtained from the Medical Faculty of the University of Cologne in May 2016 (Approval No. 16-125). Consent to publish individual patient data was obtained from participants.

Consecutive patients with an apical prolapse of the uterus or vaginal vault (Pelvic Organ Prolapse Quantification System [POP-Q] stage ≥1, Point C or D of at least −4 cm) and concurrent UUI and mixed urinary incontinence (MUI) were included in this retrospective study. Patients unsuitable for a laparoscopic procedure, those with a body mass index of >35, and those with pure SUI were excluded.

All women had undergone medical and/or conservative treatment before surgery. All patients with pure UUI received anticholinergic drugs before surgery. Most of the patients with MUI and apical POP-Q stage 1 also received anticholinergic drugs. According to the recommendations of the ethics committee, patients were informed that laparoscopic CESA or VASA (laCESA or laVASA, respectively) would be performed instead of abdominal CESA or VASA. Furthermore, patients were informed about the clinical outcomes of abdominal CESA and VASA, which were first described by Jäger and colleagues in 2012, and they were informed that limited evidence is available for the long-term outcomes of these procedures.^3,7^ Patients with vaginal vault prolapse underwent laVASA, whereas those with uterine prolapse underwent laCESA with subtotal hysterectomy.

All patients underwent preoperative and postoperative urogynecological examination at the outpatient clinic. The examination included recording the history of prolapse and UI symptoms. POP was objectively assessed using the POP-Q system described by Bump and colleagues.^9^ The objective cure rate was defined as no prolapse in the apical compartment (apical POP-Q stage 0).

Furthermore, all patients had UI, as determined on the basis of their subjective complaints rather than by urodynamic studies. All patients completed validated UI questionnaires.^10–12^ The clinical diagnoses of SUI, UUI, and MUI were based on patients' responses to the questions in the International Consultation on Incontinence Questionnaire-Short Form (ICIQ-SF) questionnaire. Thus, the clinical diagnoses were defined using question 4 of the ICIQ-SF questionnaire: “When does urine leak?” Patients were diagnosed with SUI if they had urinary leakage on coughing, sneezing, or any physical activity/exercise. Patients were diagnosed with UUI if they had urinary leakage before they could get to the toilet, and patients were diagnosed with MUI if they had both SUI and UUI.^10^ The status of patients was classified as “continent” if no symptoms of incontinence occurred postoperatively.

The primary outcome measure was the restoration of apical fixation, which was defined as apical POP-Q stage 0 at 4 months after surgery. The secondary outcome measure was the restitution of urinary continence. All patients were assessed at 2, 4, 8, and 16 weeks postoperatively in the outpatient clinic, and a year after surgery, they were contacted once a year for follow-up.

For the laparoscopic procedure, after the induction of general anesthesia, one dose of a prophylactic antibiotic was administered. Patients were maintained in the dorsal lithotomy position. An indwelling catheter was inserted into the bladder. Depending on the presence of the uterus, a vaginal probe was placed in the vagina to identify and manipulate the vaginal vault intraoperatively, if necessary. Patients were maintained in a head-down position of 25°. CO_2_ pneumoperitoneum was established according to institutional standards, and five trocars were placed. The origin and attachment of the USLs were identified at the cervix by elevating the uterus or vaginal cuff by using the vaginal probe and at the sacral vertebra by using a swab.

**FIG. 1.**
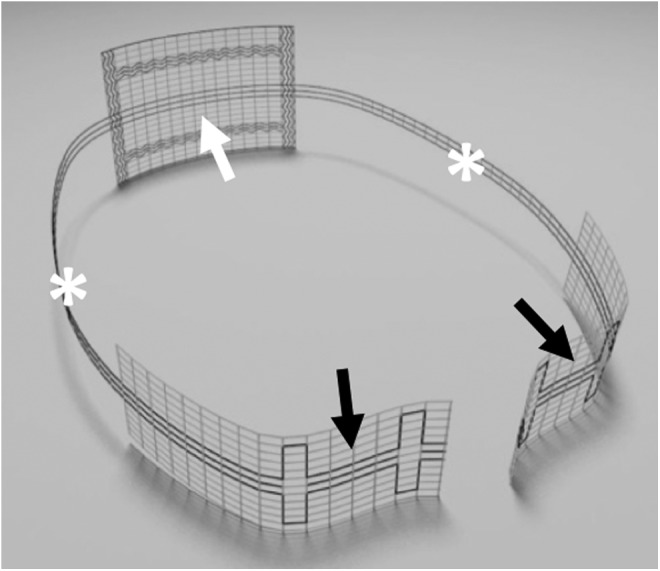
PVDF ligament-replacement structure for CESA. The *white arrow* shows the central part of the structure for fixation at the anterior cervix. The two *black arrows* show the posterior fixation sides at the left and right prevertebral fascia. The two *white asterisks* indicate the USL replacement structure on both sides of the small pelvis. CESA = cervicosacropexy; PVDF = polyvinylidene fluoride; USL = uterosacral ligament.

**FIG. 2.**
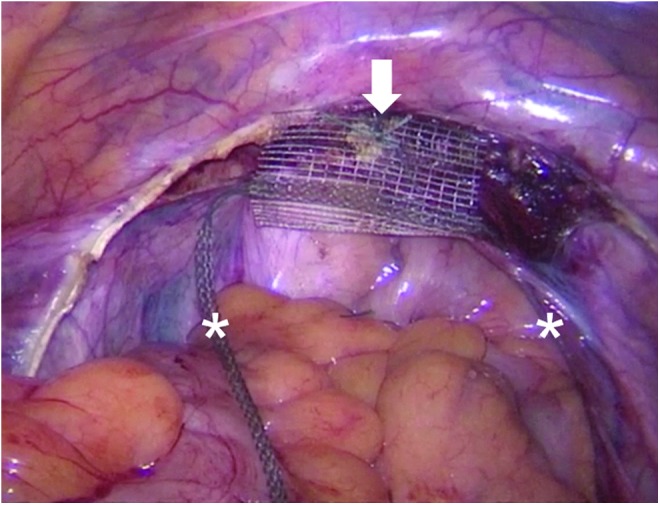
Fixation of the central part of the PVDF ligament-replacement structure at the anterior cervix with three nonabsorbable sutures (*white arrow*). Note that only one suture is shown in this figure. The two *white asterisks* mark each arm of the PVDF ligament-replacement structure for USL replacement. Note that the right part of the PVDF structure already runs below the peritoneal fold of the right USL.

Subtotal hysterectomy was performed by dissecting the uterus above the origin of the USL at the cervix. The bladder was not separated and remained on the cervix. In laVASA, the peritoneum over the vaginal vault was opened along the running scar to expose the vault. For USL replacement, a standardized PVDF ligament-replacement structure was used (Dynamesh CESA or VASA; FEG Textiltechnik mbH Company, Aachen, Germany) (Fig. 1). The central part of this PVDF structure was sutured to the cervix or vaginal vault by using three interrupted sutures. Initially, in four patients, fast absorbable sutures were used. Thereafter, nonabsorbable polyester sutures were used instead (Ethibond; Ethicon, Someville, NJ) (Fig. 2).

**FIG. 3.**
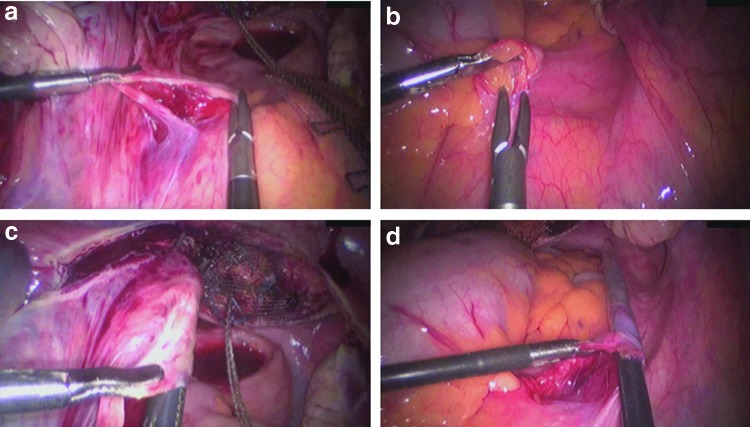
Opening of the peritoneum above the S1 sacral vertebra, and preparation of the prevertebral fascia on the left **(a)** and right **(b)**. Tunneling of the left **(c)** and right **(d)** USL toward the cervix.

**FIG. 4.**
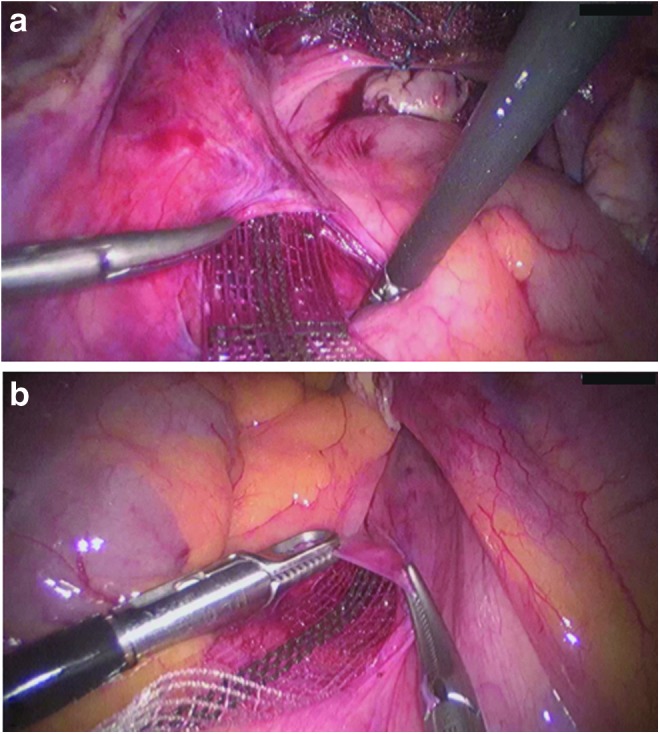
Placement of the PVDF ligament-replacement structure within the peritoneal fold of the left **(a)** and right **(b)** USL.

**FIG. 5.**
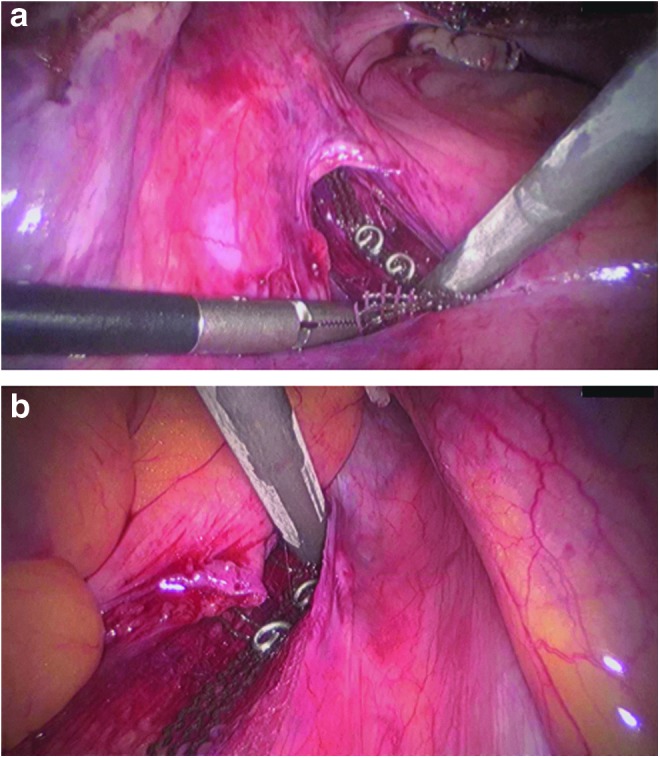
Posterior fixation of the PVDF ligament-replacement structure with a fixation device. Three titanium helices were fixed to the prevertebral fascia of the sacral vertebra at the left **(a)** and right **(b)**.

The peritoneum over the first sacral vertebra (which is the attachment site of the USL) was blunt-opened for 1.5 cm on either side of the rectosigmoid colon; consequently, the ureters and iliac vessels were located laterally, and the hypogastric nerve was spared. A clamp was inserted from the sacral peritoneal window under the peritoneum along the USL on both sides toward the cervix or vaginal vault (Fig. 3). Each arm of the PVDF ligament-replacement structure was held, and the clamp was brought back to the sacrum through the tunneled peritoneal fold of the USL on each side of the small pelvis (Fig. 4). Each arm of the PVDF ligament-replacement structure was attached with three titanium helices to the prevertebral fascia of S1 by using a fixation device (ProTack, Covidien, Mansfield, MA) (Fig. 5).

**FIG. 6.**
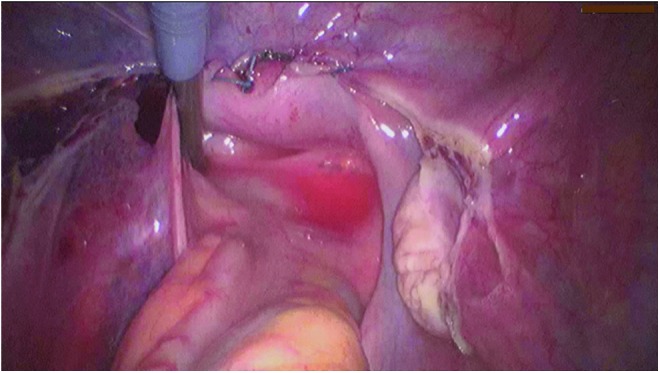
At the end of surgery, the PVDF ligament-replacement structure is all covered up with peritoneum. The flexibility of the left PVDF structure is demonstrated with an instrument.

Finally, the peritoneum above the cervix or vaginal vault was closed to cover the PVDF structure (V-Loc 180 absorbable; Covidien) (Fig. 6). For adhesion prophylaxis, a saline solution (150 mL) was poured into the abdominal cavity before removing the trocars. The indwelling catheter was removed on the day of surgery 3 hours after completing general anesthesia (Table 3).

Data collection was performed using Excel 2011 (Microsoft Corporation, Redmond, WA). Metric variables are presented as means ± standard deviations and medians, and frequencies are expressed in percentages. Statistical analyses were conducted using SPSS (Version 22; IBM, Armonk, NY).

## Results

This study included 120 women, with a median age of 66 years (Table 1). All women had UI and had POP-Q stages 1–4, with an apical descent until at least half way to the hymenal ring (POP-Q Point C or D of at least −4 cm). Table 1 presents the baseline clinical characteristics of patients. Of the 120 patients, 63 (53%) had POP-Q stage 1 apical prolapse and 57 (47%) had POP-Q stages 2–4 apical prolapse.

**Table 1.** Baseline Characteristics of the 120 Patients

*Characteristics*	*Value*^a^
Age, years	66 (30–88)^b^
Body mass index^c^	28 (18–39)^d^
Parity	2 (0–9)^d^
Pelvic organ prolapse, *n* (%)^e^	
Apical POP-Q stage 0	0 (0)
Apical POP-Q stage 1	63 (53)
Apical POP-Q stage 2–4	57 (47)
History of previous surgery, *n* (%)	
Total abdominal hysterectomy^f^	22 (18)
Total laparoscopic hysterectomy+anterior colporrhaphy	4 (3)
Vaginal hysterectomy+anterior colporrhaphy	10 (8)
Subtotal hysterectomy	1 (1)
Anterior colporrhaphy/colposuspension/pelvic floor repair/transobturator tape insertion	2 (2)

^a^ Values are given as number of all patients (percentage), unless indicated otherwise.

^b^ Values are given as median (range).

^c^ Values calculated as weight in kilograms divided by the square of height in meters.

^d^ Values are given as mean (range).

^e^ Apical prolapse according to the POP-Q system.

^f^ In 15 out of these 22 patients, a concomitant anterior colporrhaphy was documented.

POP-Q = Pelvic Organ Prolapse Quantification System.

Patients with apical POP-Q stage 1 had undergone conservative treatment, including anticholinergic drugs, or anti-incontinence surgical procedures before laparoscopic surgery. A total of 37 patients had undergone prolapse or anti-incontinence surgical procedures (Table 1). No concurrent vaginal repair or anti-incontinence surgery was performed with laCESA or laVASA. The median operation time was 88 minutes (range: 34–194 minutes). The mean hospitalization was 3 days (range: 2–5 days). No mesh erosions or obstructed defecation was detected during follow-up (Table 2).

**Table 2.** Operative Details and Complications of the 120 Patients

*Variable*	*Value*
Type of surgery, *n* (%)	
laVASA	37 (31)
laCESA	83 (69)
Concurrent hysterectomy	83
Laparoscopic subtotal hysterectomy	79
Total laparoscopic hysterectomy	4
Concurrent vaginal surgery	0
Transobturator tape insertion	Not performed
Anterior colporrhaphy	Not performed
Operating time (minutes), median (range)	88 (34–194)
Hospitalization (days), mean (range)	3 (2–5)
Complication, *n* (%)	
Bladder injuries	1 (1)
Bowel perforation	1 (1)^a^
Significant bleeding (intraoperative)	0 (0)
Reoperation for apical prolapse	4 (4)^b^
Urinary retention (within hospital stay)	1 (1)
Obstructed defecation	0 (0)
Mesh erosion	0 (0)
Conversion to laparotomy	0 (0)

^a^ Patient with severe adhesion formation of the bowel after laparotomy. Surgical revision 3 days postoperatively.

^b^ Relapse of apical prolapse within the first 2 months after surgery because of insufficient cervical fixation (fast absorbable sutures at the cervix) and relaparoscopy.

laCESA = laparoscopic cervicosacropexy; laVASA = laparoscopic vaginosacropexy.

The laparoscopic surgical techniques laCESA and laVASA were developed based on the abdominal CESA and VASA procedures described by Jäger and colleagues.^3^ Table 3 presents the main differences between abdominal and laparoscopic CESA and VASA procedures.

**Table 3.** Differences in the Surgical Steps Between the Abdominal and Laparoscopic Cervicosacropexy and Vaginosacropexy Surgical Techniques

*Surgical steps*		*Abdominal CESA and VASA*^a^	*Laparoscopic CESA and VASA*
(1)	Preoperative preparations	Bowel cleansing with 1 L CleanPrep	No bowel cleansing
(2)	Surgical access path	Head-down position 20° Pfannenstiel incision	Head-down position 25° Establishment of CO_2_ peritoneum^b^ Five trocars Umbilical (Ø 10 mm) Left lower abdomen (Ø 10 mm)^c^ Right lower abdomen (Ø 5 mm)^c^ At symphysis (Ø 5 mm)^d^ Supraumbilical (Ø 5 mm)^e^
(3)	Preparation of anterior fixation sides	Subtotal hysterectomy with monopolar electric knife	Subtotal hysterectomy with bipolar electric scissor Bilateral discontinuation of peritoneum paracervical until uterine arteries, after the USL for 2–3 cm
(4)	Anterior fixation of PVDF ligament-replacement structure	Centrally sutured to the cervix or vaginal vault with four interrupted, nonabsorbable polyester sutures	Centrally sutured to the cervix or vaginal vault with three interrupted, nonabsorbable polyester sutures
(5)	Tunneling of USL remnants	Reusable curved hook with a handle	Straight 43 cm long clamp (inserted through supraumbilical trocar)
(6)	Preparation of posterior fixation sides	Sharp incision at left and right margin of sacral vertebra at level of S1/S2	Blunt opening (1.5 cm) of peritoneum at left and right margin of sacral vertebra at level of S1/S2
(7)	Posterior fixation of PVDF ligament-replacement structure	At defined fixation sides at PVDF structure with two interrupted, nonabsorbable polyester sutures At left and right longitudinal ligament of S1/S2	Between defined fixation sides at PVDF structure with 3 titanium helices At left and right longitudinal ligament of S1/S2
(8)	Peritoneal closure	Anterior: above cervix or vault: running nonabsorbable suture Posterior: above sacral vertebra: running absorbable suture	Anterior: above cervix or vault: running suture with a nonabsorbable suture Posterior: above sacral vertebra: no closure

^a^ Described by Jäger and colleagues ^3^

^b^ According to institutional standards.

^c^ In the anterior axillary line at the level of superior spina ischiadica, lateral to the epigastric vessels.

^d^ Within the middle line 3 cm above symphysis.

^e^ For special purposes and “tunneling” of the peritoneum.

CESA = cervicosacropexy; PVDF = polyvinylidene fluoride; USLs = uterosacral ligaments; VASA = vaginosacropexy.

Four months after laCESA or laVASA, 120 (97%) patients had apical POP-Q stage 0, that is, their apical vaginal support was restored. The first four patients (3%) experienced a relapse of apical prolapse because of insufficient cervical stump fixation immediately after surgery. Furthermore, they became immediately incontinent again. In these patients, fast absorbable sutures were used. Subsequent laparoscopy with refixation at the cervix by using nonabsorbable sutures was conducted, and anatomy and urinary continence were restored in these four patients (Table 4).

**Table 4.** Pelvic Organ Prolapse Before and After Laparoscopic Cervicosacropexy and Laparoscopic Vaginosacropexy

*Clinical outcome*	*Before surgery*	*After surgery*
Pelvic organ prolapse,^a^*n* (%)		
Apical POP-Q stage 0	0 (0)	116 (97)^b^
Apical POP-Q stage 1	63 (53)	4 (3)^b^
Apical POP-Q stage 2–4	57 (47)	0 (0)

^a^ Apical prolapse according to the POP-Q system.

^b^ Relapse of apical prolapse within the first 2 months after surgery because of insufficient cervical fixation (fast absorbable sutures at the cervix) and relaparoscopy.

All 120 patients had UI before surgery; of these patients, 94 (78%) and 26 (22%) had MUI and UUI, respectively. After laCESA or laVASA, the number of patients with UI significantly reduced, and a significant difference was observed in their ICIQ-SF scores. In total, 78 (65%) patients reported continence at 4 months after surgery, whereas 42 (35%) patients reported persistent UI symptoms (Table 5). There was no significant difference in cure rates regarding the patients' POP-Q stages: 38 patients (32%) with POP-Q stage 1 (before surgery) and 40 patients (33%) with POP-Q stages 2–4 (before surgery) reported urinary continence after laCESA and laVASA.

**Table 5.** Patient-Reported Symptoms of Mixed Urinary Incontinence, Urgency Urinary Incontinence, and Pure Stress Urinary Incontinence, As Well As ICIQ Symptom Score Before and After Laparoscopic Cervicosacropexy and Laparoscopic Vaginosacropexy

	*Before surgery*	*After surgery*	p^a^
Clinical diagnoses,^b^*n* (%)			
MUI	94 (78)	34 (28)	<0.001
UUI	26 (22)	8 (7)	<0.001
Pure SUI	0 (0)	0 (0)	n.a.
Questionnaire, median (range)			
ICIQ-SF score “cured”	15 (6–21)	0 (0–3)^c^	<0.001
ICIQ-SF score “not cured”	14 (5–20)	12 (9–20)	<0.001

^a^ McNemar test and Wilcoxon signed rank test were used.

^b^ Clinical diagnoses of MUI, UUI, and pure SUI, based on patients' responses to certain questions in the ICIQ-SF (median and range).

^c^ Four patients stated leaking urine “about once a week or less often.”

ICIQ-SF = international consultation on incontinence questionnaire-short form; MUI = mixed urinary incontinence; SUI = stress urinary incontinence; UUI = urgency urinary incontinence.

Of the 94 patients with MUI, 60 (64%) achieved continence after laCESA or laVASA; of these patients, 27 had previous hysterectomy with anterior colporrhaphy. Of the 26 patients with UUI, 18 (69%) achieved continence after surgery. None of these patients had any previous vaginal surgery. Moreover, 25 of the 42 incontinent women after laCESA or laVASA underwent a secondary transobturator tape (TOT) procedure with or without anterior colporrhaphy. Of these 25 patients, 18 achieved continence after the secondary procedure.

## Discussion

Sacrocolpopexy (SCP) and sacrospinous fixation (SSF) are the standard surgical treatments for apical prolapse. However, the term “standardized” sounds confusing because the technical performance of each SCP or SSF is strongly dependant on the surgeon's own decision (including type of material, size, shape of mesh, and even positioning). The most crucial factor in this respect is the tension in the apical suspension (i.e., the lengths of these “new ligaments”), which is not defined. Accordingly, surgeons “tighten” the apex in their own way. Therefore, the comparison of clinical outcomes with respect to UI is hampered by these differences.

Because the dimensions of the bony pelvis are nearly identical among women of different ethnicities, the holding structures of the pelvic organs must also have identical lengths.^13,14^ Based on the anatomical hypotheses of DeLancey, Ulmsten, and Petros, Jäger and colleagues developed surgical techniques (CESA and VASA) for bilateral USL replacement, in which the bladder base is suspended by elevating the vaginal apex and anterior vaginal wall.^1–3^ The main difference between the CESA and VASA surgical techniques and the established techniques is the bilateral traction exerted on the vagina instead of unilateral traction. To minimize the size of the alloplastic material, the size and shape of the ligament-replacement structures were reduced to minimum (Figs. 1 and 2). Furthermore, PVDF was used to avoid the shrinkage of the USL structures, as observed with polypropylenes.^15,16^

For clinical purposes, the operation time and hospital stay are crucial. Therefore, we decided to develop CESA and VASA as laparoscopic surgical techniques. In addition to using completely different approaches and instruments, one difference between laCESA and laVASA and open abdominal CESA and VASA was the fixation of the PVDF structures at the prevertebral fascia of the S1 sacral vertebrae. We used a fixation device with titanium helices instead of sutures. Depending on the surgeon's expertise and learning curve, laCESA and laVASA can be performed in <1 hour. Patients were mobilized postoperatively on the day of surgery and were discharged 3 days after surgery (Table 2).

The effect of reduced vaginal and bladder suspension on UI is not limited to advanced prolapse, but it is also observed in minor prolapse (POP-Q stage 1).^17^ Therefore, we included patients with UI, irrespective of the stage of prolapse; accordingly, we also included patients with POP-Q stage 1. Including these patients may be peculiar because POP-Q stage 1 is considered to be the normal POP-Q stage in elderly women. However, POP-Q stage 1 covers a wide range of descents of the vaginal apex (the uterus, cervix, or vaginal vault). According to the definition of this POP-Q stage 1, point C or D can vary between −7 and −2 cm to the hymenal ring. Considering that the lengths of the female urethra may vary between 2 and 5 cm, the vesicoureteral junction was already affected in this stage of prolapse (own observation). We subdivided the results according to the patients' POP-Q stages before surgery. There was no significant difference in cure rate between patients with POP-Q stage 1 and patients with POP-Q stages 2–4. In this study, 53% patients with UI had POP-Q stage 1 (point C or D at least at −4 cm). All of these patients had undergone conservative treatment, including anticholinergic drugs, which did not restore continence in these patients. In these patients, we observed a cure rate of 69% for UUI after laCESA or laVASA. This emphasizes the importance of a properly suspended anterior vaginal wall.

Our results are consistent with those of Ludwig and colleagues and Rajshekhar and colleagues, who reported cure rates between 62% and 91% for UUI in patients with POP-Q stage 1 after abdominal CESA and VASA.^7,8^ In the aforementioned studies, apical fixation was performed using the same CESA or VASA technique as ours; thus, the difference in cure rates must be because of some additional factors. Although the reasons for the increased cure rates in this study compared with those reported by Jäger and colleagues are unknown, we assume that this difference in outcome must be attributed to prior or concomitant pelvic floor repair (e.g., anterior colporrhaphy). Additional studies should evaluate the role of this “middle compartment” for urinary continence. Because the placement of a TOT was standardized recently in addition to the standardization of CESA and VASA, the comparison of treatment outcomes between different hospitals has become feasible.^18^

## Conclusions

The CESA and VASA surgical techniques were developed as comprehensible surgical techniques for the treatment of POP. In this study, we demonstrated a laparoscopic approach with clinical outcomes. Beside the favorable anatomical outcomes, an improvement of UI was achieved too. This bilateral USL replacement was performed with minimum amount of material and a structure of defined size and shape. Furthermore, an operating time of <1 hour and a short hospitalization (mean of 3 days) are of advantage, particularly in elderly patients. Therefore, the laCESA and laVASA surgical techniques could be one treatment option for POP, especially in patients with UI.

## Supplementary Material

Supplemental data

## Abbreviations Used

CESA: cervicosacropexy
laCESA: laparoscopic cervicosacropexy
laVASA: laparoscopic vaginosacropexy
MUI: mixed urinary incontinence
POP-Q: Pelvic Organ Prolapse Quantification
PVDF: polyvinylidene fluoride
SCP: sacrocolpopexy
SSF: sacrospinous fixation
SUI: stress urinary incontinence
TOT: transobturator tape
UI: urinary incontinence
USL: uterosacral ligament
VASA: vaginosacropexy
